# Transient Bilateral Ophthalmoplegia: A Case of a Forgotten Anesthetic Medication Effect

**DOI:** 10.7759/cureus.18802

**Published:** 2021-10-15

**Authors:** Sara J Hyland, Tapan R Kavi, Nicole R Smith, Jacky Lin, Mark D Catton

**Affiliations:** 1 Department of Pharmacy, OhioHealth Grant Medical Center, Columbus, USA; 2 Department of Neurocritical Care, OhioHealth Riverside Methodist Hospital, Columbus, USA; 3 Department of Hospital Medicine, OhioHealth Grant Medical Center, Columbus, USA; 4 Department of Anesthesia, OhioHealth Grant Medical Center, Columbus, USA

**Keywords:** code stroke, ophthalmoplegia, general anesthesia, scopolamine, stroke mimic, eye ptosis, gaze palsy, adverse drug effect, nerve block

## Abstract

A 58-year-old woman was found to have bilateral ptosis and downward gaze deviation immediately after elective shoulder surgery with general anesthesia and supraclavicular nerve block. A code stroke was activated due to concern for the neurologic process, but neuroimaging did not reveal acute changes or vascular abnormality. Her symptoms gradually resolved in the following hours with supportive care and were ultimately deemed to be related to anesthetic and transdermal scopolamine exposures layered upon her underlying comorbidities. Transient bilateral ophthalmoplegia after general anesthetics has been previously described; drug effect should be considered in the differential of this alarming presentation, which can mimic acute stroke and/or Horner syndrome.

## Introduction

We present a case of new-onset extraocular movement abnormalities discovered immediately after elective shoulder surgery with general anesthesia and supraclavicular nerve block. Acute neurologic workup with computed tomography (CT) of the head and angiogram did not reveal any abnormalities and her symptoms, fortunately, were transient as they resolved in a few hours. We believe these symptoms were a medication effect of the anesthetics and transdermal scopolamine she was exposed to, superimposed upon her underlying chronic conditions. Our review of the literature revealed that transient external ophthalmoplegia may be unexpectedly common after general anesthesia, occurring in 46% of patients in one study [1]. Additionally, transient microvascular ischemic third nerve palsy is possible in this type of patient and may be more common than previously thought [2-4]. Furthermore, oculomotor function is heavily dependent upon cholinergic innervation, and common perioperative anticholinergic medications could have measurable impacts on eye function in susceptible patients [5,6]. This case promotes increased awareness of ophthalmological effects of common anesthetic medications among perioperative providers in order to support accurate diagnosis and avoid unnecessary workup or high-risk therapies when this type of presentation is encountered. This study was approved by the OhioHealth Institutional Review Board after written informed patient consent and Health Insurance Portability and Accountability Act (HIPAA) authorization was obtained. This study adheres to the case report reporting guidelines (CARE) [7].

## Case presentation

A 58-year-old woman who weighs 87.5 kg presented for revision to reverse left total shoulder arthroplasty. Her past medical and surgical histories were significant for type II diabetes mellitus, obstructive sleep apnea (non-dependent on positive airway pressure ventilation), migraines, anxiety, osteoarthritis, postoperative nausea and vomiting, antithrombin III deficiency with prior deep venous thromboses, thyroid cancer, left reverse total shoulder arthroplasty, and left blepharoplasty approximately two years prior for lifelong left-sided ptosis. 

In the preoperative area, she received routine preemptive oral analgesics (acetaminophen 975 mg, gabapentin 300 mg), application of a scopolamine patch to the right mastoid for postoperative nausea and vomiting (PONV) prevention, and intravenous fentanyl 100 mcg and midazolam 2 mg to facilitate placement of left supraclavicular nerve block by the attending anesthesiologist. The block was placed under ultrasound guidance with a total volume of 30 mL local anesthetic (10 mL of 0.5% ropivacaine and 20 mL of 2 % lidocaine, both without epinephrine). She was taken to the operating suite where she was provided general anesthesia induction with intravenous propofol 200 mg and succinylcholine 140 mg then maintenance with inhaled sevoflurane and intravenous rocuronium 50 mg. She received routine intravenous antiemetics (ondansetron 4 mg and dexamethasone 4 mg) and prophylactic antibiotics (intravenous cefazolin 2 g and topical vancomycin 1000 mg onto the surgical site) throughout an uneventful operative course of approximately two hours. The operation was pursued via the prior reverse total shoulder arthroplasty incision site with the patient in a supine "beach chair" position and included removal and replacement of all prior prosthetic components. At the end of the case, a qualitative train-of-four measurement yielded 4/4 twitches and she was administered intravenous neostigmine 3 mg with glycopyrrolate 0.6 mg. She was successfully extubated and transferred to the post-anesthesia care unit (PACU). 

**Figure 1:**
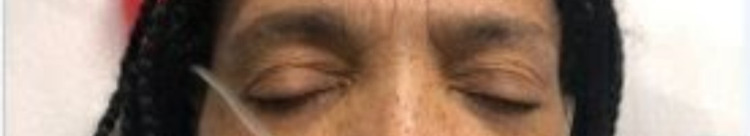
Patient exhibiting bilateral ptosis immediately after surgery

**Figure 2:**
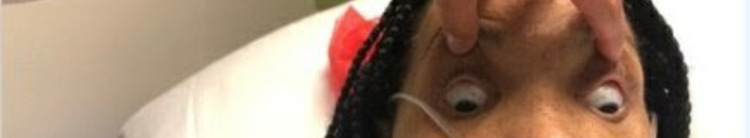
Patient exhibiting downgaze deviation immediately after surgery A medical provider is holding the patient's eyelids open to demonstrate.

Upon interview by the admitting hospitalist in the PACU, approximately 20 minutes after PACU handoff by the anesthesia providers, the patient was noted to have new-onset bilateral ptosis (left initially greater than right), bilateral downward gaze deviation, and inability to open her eyelids or move her eyes vertically or horizontally (Figures 1, 2). Her pupils were difficult to assess due to the significant downward gaze and dark iris coloration. The patient was appropriately drowsy but completely responsive and also endorsed an acute onset holocephalic headache. Physical examination was also noteworthy for left-side diminished facial sensation to light touch, though she reported this to be intermittent and chronic for her. Her vital signs remained stable and within normal limits, and the physical examination was otherwise unremarkable and appropriate for immediate postoperative shoulder surgical status. 

The attending anesthesiologist was called to evaluate the patient at this time and a stroke alert was also activated due to concern for acute neurologic process. Acute neurologic processes such as ischemic stroke, Horner’s syndrome, and others were considered but were ruled out based on bilaterality of symptoms and after reviewing emergent CT scan and CT angiogram. As per vascular neurologist recommendation, supportive care was started for possible migraine with intravenous valproic acid 500 mg and crystalloid maintenance fluid. The scopolamine patch was removed for possible anticholinergic cause of her symptoms as per clinical pharmacist recommendation. 

**Figure 3:**
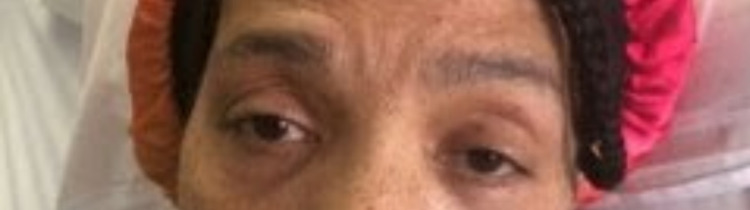
Patient back to baseline on morning of the first postoperative day

The patient’s symptoms gradually improved in the following evening hours and were noted to be completely resolved the next morning (Figure 3). A magnetic resonance imaging (MRI) scan was completed postoperative day one and revealed mild chronic white matter microvascular ischemic changes without evidence of acute intracranial abnormality. The inpatient neurology service evaluated the patient and felt the patient’s previous symptoms were likely explained by migrainous syndrome and/or anesthetic drug effect. The patient’s hospital course was also complicated by acute kidney injury, though this also resolved quickly with supportive care and she was discharged home in good condition on postoperative day two. 

The patient was unable to follow up with the neurology clinic after hospital discharge from this encounter for further testing or evaluation. She did have a successful outpatient podiatric surgery several months later, however, during which she was exposed to some of the same medications (cefazolin, general anesthesia with propofol but without paralytics or inhaled anesthetics, local lidocaine infiltration, intravenous dexamethasone, routine opioids). A history and physical examination completed at pre-admission testing for this procedure were unremarkable and noted her pupils to be equal and reactive to light. She had an uneventful surgical and anesthetic course without mention of ocular aberrations and was discharged home in good condition from the PACU at that time. A phone interview completed by the study investigators, with patient permission, suggested that the patient had not experienced ophthalmoplegia at any point prior to her revision shoulder surgery admission nor had she experienced any recurrence afterward.

## Discussion

The differentials for this acute presentation include Horner syndrome, acute stroke with vertebrobasilar syndrome, myasthenic crisis, ophthalmoplegic migraine, and anesthetic effect (persistent partial neuromuscular block in the peri-operative period). Horner syndrome is characterized by unilateral ptosis, miosis, and anhidrosis resulting from disruption of sympathetic innervation [8]. Though this has been reported with supraclavicular block, we felt it was unlikely given the patient’s bilateral symptoms and complete ptosis [9]. Acute vertebrobasilar syndrome and resultant ischemic stroke were also considered, but as imaging revealed patency of posterior circulation and as the patient had preserved extremity function, this was considered unlikely. The brain MRI later in the course showing absence of any ischemic injury also supports an etiology other than acute stroke. Myasthenic crisis can lead to ophthalmoplegia, but this also seemed very unlikely without any medical history or previous symptoms and also considering her complete ophthalmoplegia rather than characteristic fatigability. Ophthalmoplegic migraine was also considered and she was given relevant treatment with intravenous fluids and valproic acid, but the bilateral nature of symptoms and no prior similar symptoms make this unlikely as well. Moreover, this is usually seen in younger patients and our patient demographic would not be typical. Acute Wernicke’s syndrome with thiamine deficiency can also cause ophthalmoplegia but rapid improvement in symptoms without thiamine replacement suggests otherwise. The cranial variant of Guillain-Barre syndrome known as Miller-Fisher syndrome is characterized by extraocular muscle and facial muscle involvement, however, the sudden nature and lack of facial involvement in our patient along with quick recovery go against it [10]. A transient microvascular ischemic ocular nerve palsy is possible, especially given the patient’s medical risk factors. However, given the bilateral nature of her symptoms, involvement of extraocular muscles supplied by multiple cranial nerves, and prompt recovery, it was also considered unlikely [2-4]. Parinaud syndrome as an anesthetic complication was also considered [11]. Due to her restriction of eye movement in both horizontal and vertical directions as opposed to just vertical, Parinaud was considered unlikely. After pursuing an exhaustive review of pathophysiological differentials and assessing the temporal sequence of events, an anesthetic medication effect seemed like the most likely cause of the symptoms in our patient.

A literature search revealed that transient external ophthalmoplegia may be unexpectedly common after intravenous anesthesia. A prospective case series by Marsch and Schaefer evaluated eye-opening and movement functions immediately after recovery from total intravenous anesthesia with propofol, fentanyl, and atracurium [1]. Out of the 110 patients in the study, all of which had normal eye and eyelid mobility prior to induction of anesthesia, 51 patients (46%) either had the complete inability to open their eyes or showed some impairment of eye-opening and/or eye movement immediately following the operation. A total of 21 patients (19%) exhibited a complete inability to open their eyes combined with a total gaze paresis. The ophthalmoplegic symptoms were bilateral, did not affect vision, and proved transient; by the time 20 minutes had passed, almost all patients had fully recovered eye function. The authors were unable to make a determination if one specific medication caused the ophthalmoplegia, but suggest that one or more drugs may have selectively affected the supranuclear centers in the pontine and midbrain reticular formation, given that both horizontal and vertical eye movements were affected bilateral and synchronously [1]. Our patient experienced a similar presentation of bilateral external ophthalmoplegia and total gaze paresis, though symptoms resolved more gradually over the first few postoperative hours.

The patient’s transdermal scopolamine exposure may also have contributed to her symptoms since cholinergic pathways play a role in oculomotor function [5,12]. Central anticholinergic medications such as scopolamine can impair eye movements in healthy individuals due to decreased cholinergic transmission, in addition to the better-known effects of mydriasis and increased intraocular pressure [5,6,13-16]. Anticholinergic activity may not completely explain her extraocular symptoms, however, so we concluded her presentation was likely a combination of her anesthetic and anticholinergic drug exposure to which her mild chronic white matter microvascular ischemia may have rendered her susceptible. Another possible differential is unmasking of subclinical ocular myasthenia gravis by administration of rocuronium and succinylcholine especially given the patient's history of ptosis. Another possible differential is unmasking of subclinical ocular myasthenia gravis by administration of rocuronium and succinylcholine, especially given the patient's history of ptosis. This theory is plausible given her spontaneous recovery, which could be due to the paralytic drug effect wearing off. The patient did receive neostigmine which should reverse the effects of neuromuscular blockade by the paralytic agents mentioned above, however, it is possible that administration of an agent such as sugammadex may have been more effective at reversal than neostigmine. Also, given the lack of progression of symptoms and spontaneous recovery, testing for acetylcholine receptor antibodies for a diagnosis of ocular myasthenia gravis would not be indicated.

## Conclusions

In conclusion, acute postoperative onset of bilateral ptosis and extraocular movement abnormalities with spontaneous downward gaze deviation caused initial concern for an emergent neurologic pathology such as an ischemic stroke but proved transient and likely medication-related in a vulnerable patient. The likely causes in this patient being anesthetic effects on midbrain and pontine gaze centers, anticholinergic effects of scopolamine, or unmasking of subclinical ocular myasthenia gravis by administration of succinylcholine and rocuronium. Transient bilateral ophthalmoplegia after anesthesia and paralytics may be common but easily overlooked in the immediate recovery phase of care. Increased understanding of the ocular effects of common anesthetic medications can be valuable in avoiding the dangers and costs of an incorrect stroke diagnosis.
